# Manufacture of Multilayered Artificial Cell Membranes through Sequential Bilayer Deposition on Emulsion Templates

**DOI:** 10.1002/cbic.202100072

**Published:** 2021-03-31

**Authors:** Tsoi Ip, Qien Li, Nick Brooks, Yuval Elani

**Affiliations:** ^1^ Department of Chemistry Imperial College London Molecular Sciences Research Hub White City London W12 0BZ UK; ^2^ Department of Chemical Engineering Imperial College London South Kensington London SW7 2AZ UK

**Keywords:** artificial cells, droplet technologies, membrane biophysics, molecular bioengineering, vesicles

## Abstract

Efforts to manufacture artificial cells that replicate the architectures, processes and behaviours of biological cells are rapidly increasing. Perhaps the most commonly reconstructed cellular structure is the membrane, through the use of unilamellar vesicles as models. However, many cellular membranes, including bacterial double membranes, nuclear envelopes, and organelle membranes, are multilamellar. Due to a lack of technologies available for their controlled construction, multilayered membranes are not part of the repertoire of cell‐mimetic motifs used in bottom‐up synthetic biology. To address this, we developed emulsion‐based technologies that allow cell‐sized multilayered vesicles to be produced layer‐by‐layer, with compositional control over each layer, thus enabling studies that would otherwise remain inaccessible. We discovered that bending rigidities scale with the number of layers and demonstrate inter‐bilayer registration between coexisting liquid–liquid domains. These technologies will contribute to the exploitation of multilayered membrane structures, paving the way for incorporating protein complexes that span multiple bilayers.

## Graphical abstract

**Running rings around vesicles**: We describe a droplet‐based technology to manufacture cell‐sized multilamellar lipid vesicles. Individual membranes are assembled layer‐by‐layer, giving control over the number of induvial layers and their molecular content. We investigated how the biomechanical properties of the structures change with each added layer.

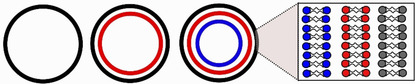

## Introduction

One of the grand challenges of our times is to design and construct entirely artificial cells from the bottom up using biomolecular building blocks. Mimicking the architectures, processes, and behaviours found in biological cells allows researchers to investigate phenomena in cell biology using simplified cell models through a “learning by building” approach.^[1]^ Engineering artificial cells as micromachines that are programmed to perform useful tasks also has a tremendous number of potential applications in clinical, biomedical, and industrial settings.^[2]^

One cellular structure in particular has proved particularly suited to this reductionist approach: the cell membrane. Wide and diverse insights into membrane properties have been elucidated using model membranes, including the role of curvature,^[3]^ fluidity,^[4]^ viscosity,^[5]^ phase separation,^[6]^ elasticity,^[7]^ and asymmetry^[8]^ in cell biology. Model membranes are also heavily used in the study and biotechnological exploitation of both integral and membrane‐associated proteins.^[9]^ They are also increasingly being leveraged in the design of smart therapeutic delivery systems, responsive microreactors, and sensors.^[10]^

Artificial cell membranes often take the form of enclosed phospholipid bilayers that adopt a quasi‐spherical shape, known as vesicles or liposomes. For artificial cell applications these are typically 1–100 μm in diameter (cell‐sized), and are composed of a single bilayer (giant unilamellar vesicles; GUVs). However, this model may, in certain instances, be deficient. There are several membranous motifs that consist of multiple bilayers, including bacterial double membranes and the membranes surrounding the nucleus, chloroplasts, and mitochondria. In these cases, each bilayer in the multilayered construct has a distinct molecular composition.^[11]^ Multilamellar structures also form part of the endoplasmic reticulum, Golgi apparatus, lamellar bodies, and the thylakoid membranes of photosynthesising organisms and organelles.

A technological bottleneck exists which prevents such multi‐membrane constructs from being manufactured. This has hindered our ability to create appropriate models with which to investigate the biophysics and biochemical roles of multilayered membranes, and prevents reconstitution of protein assemblies that span multiple bilayers (e. g., the nuclear pore complex,^[12]^ protein secretion systems,^[13]^ bacterial flagella,^[14]^ cellulose extrusion system).^[15]^ Furthermore, it has stymied progress in realising some of the applications for multilayered membranes, including in gene delivery, photonics, and bio‐inspired electrical devices.^[16]^ Herein, we overcome this hurdle by developing a layer‐by‐layer biomembrane engineering technology.

The dominant method to generate vesicles for synthetic biology is emulsion phase transfer, which converts water‐in‐oil droplets into vesicles.^[17]^ This has been an established method for forming unilamellar (single‐layered) vesicles,^[18]^ and has been at the heart of many artificial cell advances due to its high encapsulation efficiency of large, charged biomolecules, including proteins, DNA, and macromolecular complexes. This is a prerequisite for vesicles to be used as artificial cell chassis, and is in contrast to more traditional methods for GUV generation (e. g., gentle hydration, electroformation, solvent extraction) which have low encapsulation efficiencies.^[19]^

Although some existing vesicle generation methods can result in stochastic production of some multilamellar structures, the number and molecular composition of each bilayer cannot be controlled.^[18]^ A recent microfluidic method has partly addressed this issue;^[20]^ however, although powerful, this method has low throughput (<15 vesicles per experiment), rendering it unsuitable for mass production as well as requiring cleanroom infrastructure, extensive resources and training.

In this work, we detail a technology which allows individual bilayers to be assembled one‐by‐one around emulsion droplets, which in turn allows single‐, double‐, and triple‐layered vesicles to be manufactured with control over the molecular composition of each layer. We validate the presence of multiple bilayers, and demonstrate how this technique can be used as an experimental tool with which to study some long‐standing hypotheses in membrane biophysics. We show that the multiple lamellae are mechanically coupled, with the bending rigidity of the multi‐layered membrane as a whole scaling with the number of bilayers present. We also demonstrate that coexisting liquid‐liquid phase‐separated domains align with each other through multiple bilayers.

## Results and Discussion

### Multilayered vesicle generation

**Figure 1:**
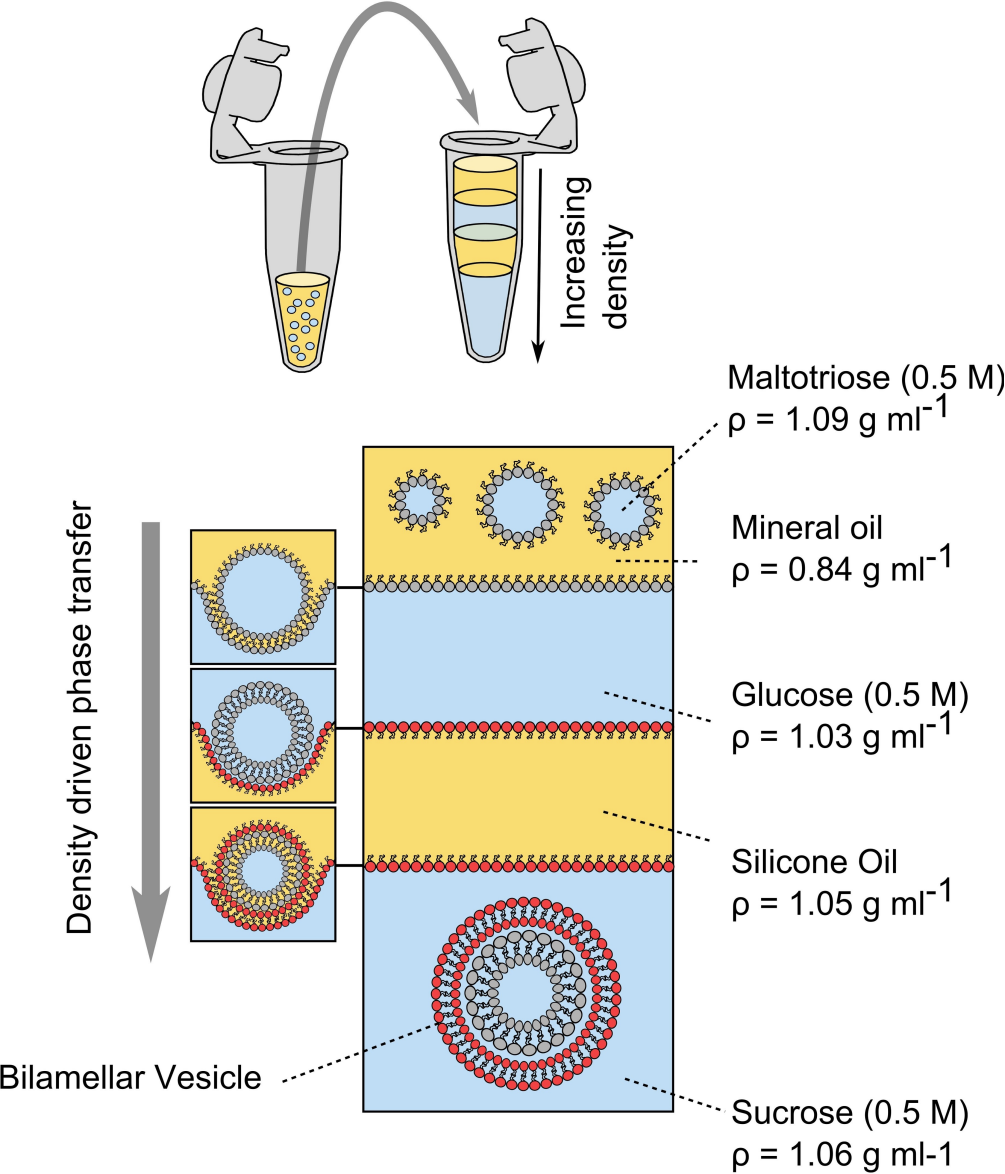
Schematic of the two‐layered vesicle generation setup. Lipids are dissolved in the oil phases and self‐assemble at all water/oil interfaces. A stable multilayered water/oil/water/oil column is generated by ensuring each layer is denser than the one above it. Water‐in‐oil emulsion droplets, which are the densest solution in the system, are added to the top of the column. These are driven through the interface under centrifugation, picking up sequential monolayers to generate bilamellar vesicles at the bottom. Three‐layered vesicles can be formed in a two‐step process by performing an additional centrifugation round.

Our technology uses water‐in‐oil droplets as templates around which sequential lipid monolayers are deposited (Figure 1). In our setup, we dissolve the lipid in the oil phases, and have a series of water/oil interfaces stabilised by a lipid monolayer due to their amphiphilic nature. The innermost monolayer is formed by incubating aqueous emulsion droplets in an oil solution. Subsequent monolayers are deposited by driving the droplet through several monolayer‐stabilised oil/water interfaces. Each time the droplet crosses an interface, another monolayer is deposited. This allows us to form one‐, two‐, or three‐layered vesicle membranes which encapsulate the content of the original emulsion droplet.

We used density differences to drive droplets through the interfaces. This required us to make a multilayered column consisting of alternating water and oil phases, where each layer is denser than the one above it. Lipid‐stabilised emulsion droplets, which have the largest density in the system, are added to the top of the column, which enables them to be driven through the column when placed in a centrifuge, leading to the manufacture of multilayered vesicles. Appropriate densities are achieved by using different oils (mineral oil, silicone oil AR 200; *ρ*=0.84 g mL^−1^, 1.05 g mL^−1^ respectively) and dissolving different sugars in the aqueous phases (0.5 M glucose, sucrose, maltotriose; *ρ*=1.04, 1.07, 1.08 g mL^−1^ respectively).

To generate two‐layered vesicles, the column consisted of water/oil/water layers, with a water‐in‐oil emulsion added to the upper aqueous phase. To generate three‐layered vesicles, this process was repeated with previously prepared two‐layered vesicles deposited above a further water/oil/water column. Importantly, the lipid composition of each oil phase corresponds to the composition of the bilayer that is being assembled, allowing the construction of vesicles with a defined molecular composition in each layer.

**Figure 2:**
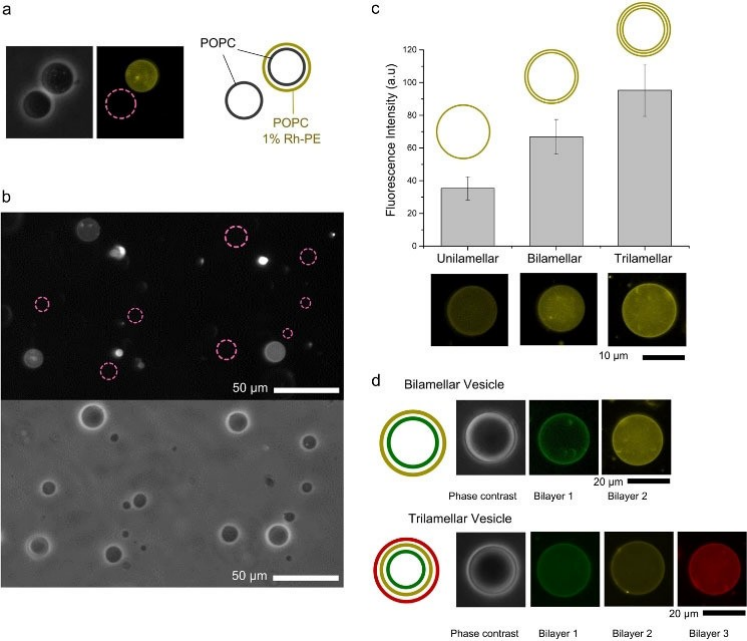
Multilayered vesicles. a) Phase contrast (grey) and fluorescence (yellow) images of one‐ and two‐layered vesicles. Purple *′Pink dashed′* circles represent vesicles that are not visible under fluorescence mode, and hence do not contain the external bilayer. b) Fluorescence (above) and phase contrast (below) images of a population of one‐ and two‐layered vesicles. c) Average fluorescence intensity of one‐, two‐, and three‐layered vesicles; error bars=S.E., *n*=20. d) Fluorescence images of two‐ and three‐layered vesicles with different fluorescently labelled lipids (1 %) in each leaflet. Dyes used are NBD‐PE (green), Rh‐PE (yellow), and Cy‐5 (red).

Throughout our experiments, when vesicles successfully had multiple membranes deposited, this was identified by incorporating a fluorescently labelled lipid (1 % Rh‐PE or NBD‐PE) to the external membrane only, by adding it to the silicone oil layer. The presence of a fluorescence signal in the vesicle membrane was taken as an indicator that multiple membranes were assembled (Figure 2a and b). Non‐fluorescent vesicles were assumed to be those in which the external layer was not successfully deposited. 26 % (*n*=850) of vesicles were found to be two‐layered, and vesicles were cell sized (0.5–20 μm radius). When vesicles were taken through a further centrifugation round to produce three‐layered vesicles, the yield reduced to 6 % (*n*=450). By counting the number of multilayered vesicles per well of a defined volume, we estimate that the number generated using our approach is in the order of 5 x 10^4^ cell‐sized vesicles per 1 mL Eppendorf batch. We note that the individual bilayers could not be seen in the phase contrast images as the size of the inter‐membrane space is below optical resolution.

### Validation of multilayered membranes

Successful multilayered vesicle generation was confirmed by generating one‐, two‐, and three‐layered POPC vesicles with 1 % Rh‐PE fluorescent lipid in each layer and analysing their fluorescence intensities. As expected, the addition of each layer led to a corresponding increase in fluorescence in approximate integer multiples (Figure 2c). Furthermore, control over the composition of each layer was demonstrated by added different fluorescent lipids to each oil phase that corresponded to each layer (NBD‐PE, Cy‐5‐PE, and Rh‐PE), with corresponding fluorescence signals appearing in two‐ and three‐layered vesicles (Figure 2d).

**Figure 3:**
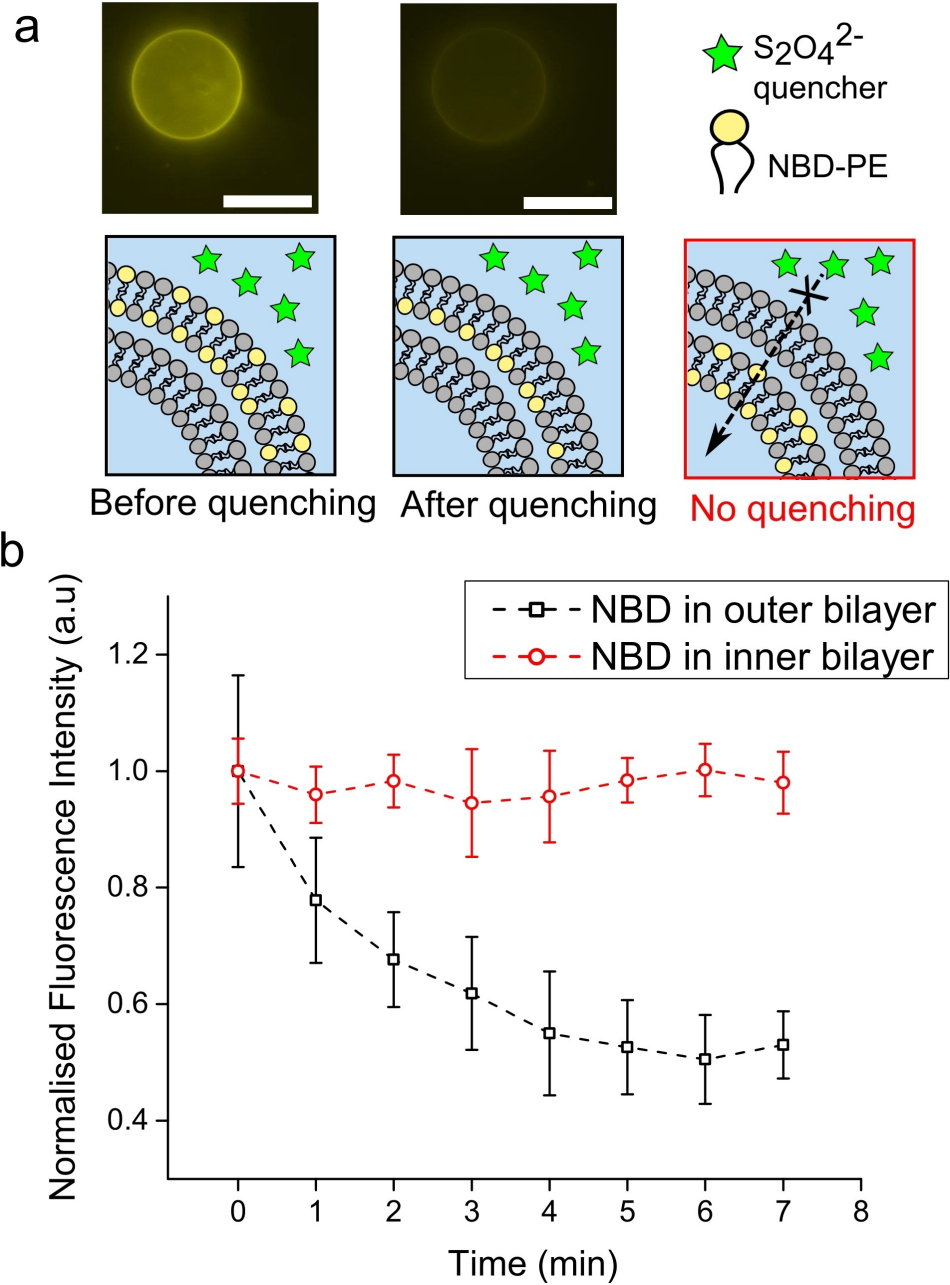
Validation of the presence of two bilayers in a quencher assay. a) The membrane‐impermeable S_2_O_4_^2−^ quencher was added to a solution of two‐layered vesicles, where the external bilayer contained 1 % NBD‐PE fluorescent lipid. In the control scenario, NBD‐PE was in the inner bilayer instead. Fluorescence microscopy images are shown before and after quenching. Scale bars: 5 μm. b) Graph showing average fluorescence signal of the vesicle boundary. An approximate 50 % decrease was seen when NBD‐PE was present in the external bilayer, as only one monolayer leaflet was exposed to the quencher. Error bars correspond to standard error, n=20.

The presence of multiple layers was further validated using an assay involving a fluorescence quencher present in the external solution (Figure 3). Two‐layered vesicles were generated with NBD‐PE present in either the inner or outer bilayers. Sodium dithionite (0.05 M), which is a membrane‐impermeable NBD quencher, was added the vesicle exterior, and vesicles were imaged using fluorescence microscopy. Two‐layered vesicles with NBD in the inner layer retained their fluorescence, as the external membrane protected the inner membrane from exposure to the dye. There was no significant difference compared to the control scenario in which no quencher was added (unpaired t‐test, *p*<0.001, *n*=20). Conversely, when NBD was present in the external bilayer only, fluorescence was quenched. A 47 % reduction (S.E.=5.3 %, *n*=20) of fluorescence was observed, as only the external facing leaflet of the bilayer was exposed to the quencher. A time‐course experiment, where two‐layered vesicles were imaged immediately after quencher dye was added, revealed that quenching began almost immediately and was completed within 5 minutes (Figure 3). These experiments also confirm compositional control of each layer in the multilamellar assembly. We note that in the above experiments, only two‐layered vesicles were analysed; as before, these were differentiated from single‐layered vesicles in the population through the presence of a fluorescently labelled lipid in the outer bilayer.

### Biophysical characterisations

Biophysical properties of membranes, such as tension, stored curvature elastic stress, lateral pressure profile, and viscosity are now recognised as playing significant roles in cellular processes. Membrane mechanics, including the bending rigidity (the energy required to bend a membrane of defined area away from its equilibrium position), have been shown to influence membrane protein folding,^[21]^ stability,^[22]^ activity,^[23]^ and gating,^[24]^ and have been implicated in homeostatic control^[25]^ and parasite/host interaction.^[26]^ It has been proposed that multilayered membranes have bending rigidities that scale in relation to the number of layers present.^[27]^ However, due to a lack of methods generate multilayered membranes, this has not been systematically experimentally validated.

**Figure 4:**
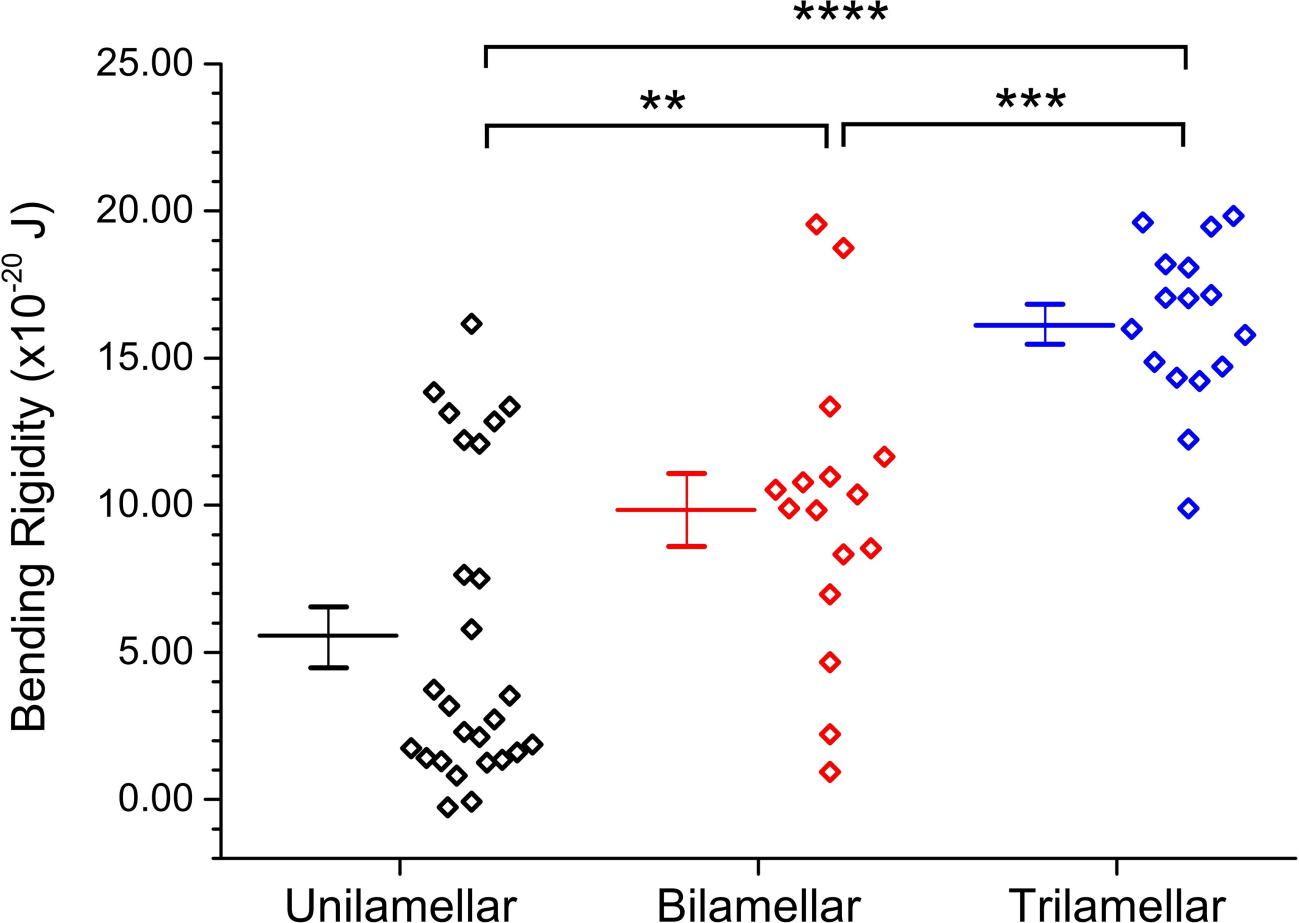
Bending rigidity of multilayered vesicles. Each data point corresponds to values obtained from distinct vesicles. Solid lines represent the mean. P values calculated using an unpaired t‐test (** p<0.01, *** p<0.001, **** p<0.0001).

Our method enabled us to use flickering analysis to determine the bending rigidity of single‐, double‐, and triple‐layered membranes. This is a non‐invasive method where thermal fluctuation of vesicles are detected using phase‐contrast microscopy.^[28]^ Shape fluctuations away from the equilibrium form are monitored and the mean square values of the deviations are determined. This analysis revealed statistically significant differences between single‐, double‐, and triple‐layered vesicles (Figure 4), with values for unilamellar vesicles being in the same range of those found in literature.^[29]^ Increasing the number of layers led to an increase in the bending rigidity, with values being approximate integer multiples of those associated with single‐layered vesicles, confirming that the bilayers are mechanically coupled to one another. Differences in bending rigidity were found despite the large scatter in the data, which we attribute to a combination of vesicle‐to‐vesicle variations in both the level of residual oil in the membranes and inter‐membrane distance, as well as inherent noise associated with the flickering spectroscopy data capture and analysis.

### Domain alignment

Lipid bilayers consisting of multiple lipid types can possess coexisting domains of phases with different ordering, for example a liquid‐ordered (L_o_) and liquid‐disordered L_d_ phase.^[30]^ It is well known that in bilayer membranes, domains in opposite monolayer leaflets align due to trans‐bilayer coupling.^[31]^ A lack of methods to controllably generate multilamellar vesicles has limited investigations into possible alignment (or registration) of domains between multilamellar vesicle bilayers (as opposed to between two monolayers of a bilayer). Here, we demonstrate the potential of our technology to address this.

**Figure 5:**
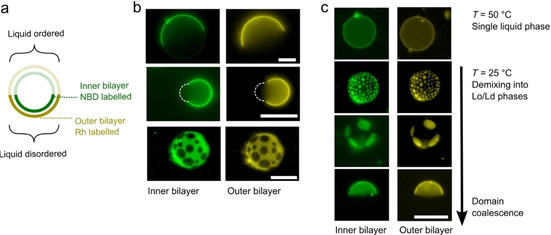
Domain alignment in two‐layered vesicles. a) Schematic showing inter‐bilayer domain alignment in a two‐layered ternary mixture with vesicles exhibiting liquid‐liquid phase separation. b) Fluorescence images of inner and outer bilayers of two‐layered vesicles including spherical (top), bulging (middle), and multi‐domain vesicles (bottom). Dotted white lines represent bulging areas of the vesicle, not visible under fluorescence. Domains are aligned in all cases. c) Domains remain aligned through the demixing process, when vesicle were cooled below their miscibility temperature, from a fully mixed single liquid regime to a phase‐separated one. Vesicles were composed of DPhPC/EggSM/cholesterol 1 : 1 : 3. Scale bars: 20 μm.

We generated two‐layered vesicles which had a ternary lipid composition (DPhPC/EggSM/cholesterol 1 : 1 : 3) that results in coexisting L_o_/L_d_ domains at room temperature.^[32]^ We added 1 % NBD‐PE lipid to inner membranes and 1 % Rh‐PE to the outer ones. These dye‐labelled lipids both preferentially segregate into the L_d_ phase, allowing us to visualise phase separation. We found that there was complete domain alignment between the bilayers, including in vesicles that bulged away from a spherical geometry and those which had a multidomain morphology (Figure 5b). Indeed, when domains were taken past their miscibility temperature to 50 °C to produce a uniform liquid phase, and then brought back down to room temperature, domains remained in alignment throughout the whole process (Figure 5c). We did not observe domain misalignment during the demixing transitions, indicating that alignment equilibrium is reached at timescales below those which can be detected using our setup (ca. 0.5 s). These results agree with experiments and models that demonstrate that domain alignment occurs in multilamellar lipid stacks composed of hundreds of bilayers,^[33]^ possibly due to surface tension effects arising from the network of hydrogen‐bonding water molecules that reside in the intermembrane space. We note the composition given is the composition of the lipid film that was dissolved in the oil, and is not the final composition of the membranes; studies of analogous oil‐based methods of manufacturing vesicles have shown that cholesterol incorporates at lower efficiencies, at approximately 30 % of the level compared to phospholipids.^[34]^

## Conclusion

Our method allows the manufacture of multilayered vesicles where the number of bilayers, and their lipid content, can be defined. This allowed us to determine that the membrane bending rigidity scales with the number of layers added, which also confirms that individual bilayers are mechanically coupled. Because each layer changes the biophysics of the ensemble in defined increments, this raises the possibility of manufacturing designer vesicles with tuneable properties (e. g., permeability, mechanical strength, elasticity, release profiles) which could be important for applications such as drug delivery and storage of bioactives.^[35]^ In contrast to other approaches, this is a bulk method, which paves way for the mass production of multilayered vesicles, which is a prerequisite for their deployment in biomedical applications and in biotechnology more generally, as opposed to fundamental science where single‐vesicle analysis is often sufficient.

We also demonstrated alignment of coexisting domains between individual bilayers. This is a key finding, as in biological membranes phase‐separated domains are thought to influence cellular events, such as signalling and sensing, by recruiting and co‐localising membrane proteins. If coupling occurs *between* individual bilayers in a multilamellar assembly, this could have biological consequences, for example leading to alignment of proteins between inner and out membranes. Trans‐bilayer coupling could offer a mode of communication between receptors on multiple bilayers of a multimembrane structure, and may form part of a multimembrane signal transduction pathway. A similar phenomenon is indeed thought to occur in monolayer coupling between bilayer leaflets.^[36]^ Taken together, the results above demonstrate the wealth of biophysical studies that can be unlocked using this approach, which have to date remained inaccessible.

We note that with further technological developments, some improvements of the described platforms could be made. Firstly, the yield of successful deposition of a further bilayer on a GUV is below 30 %, and there are a lack of available methods to purify these vesicles from the rest of the population. Secondly, our method requires correct differences in density between the different aqueous and oil phases in the column. This may place a limitation on the content one may wish to have in the inner and outer aqueous phases, and may limit the choice of oils that can be used in this setup. Similarly, as this method relies on lipids first being dissolved in the oil phase, only oil‐soluble amphiphilic building blocks can be used. Thirdly, there exists the possibility of residual oil being present in the bilayers, which may have an effect on biomembrane mechanics and transmembrane protein function. Finally, the vesicle size distribution is polydisperse, which may be problematic for certain applications.

These results feed into the artificial cell endeavour, as much of the biological machinery that is critical for imbuing cells with “behaviours”, such as motility, biopolymer extrusion, energy generation and signal transduction into cells span multiple membranes. Not only will multi‐membrane constructs facilitate the incorporation of behavioural modules into artificial cells for biotechnological applications (e. g., for therapeutic delivery and bioreactors), but it may also aid in the understanding of core biological processes in a model environment, for example by incorporating double‐ or triple‐membrane‐spanning protein complexes in an appropriate synthetic scaffold.

Realising this potential will be aided by further developments, including technologies to scale down the size of the constructs to the approximately 200 nm regime required for drug delivery applications, purification of the multilamellar constructs for the rest of the vesicle population, and increasing the number of deposited bilayers (to tens or even hundreds of layers). The use of microfluidic devices may offer potential solutions to these challenges.^[37]^

## Experimental Section

**Vesicle generation**: All lipids were purchased from Avanti Polar Lipids, and reagents from Sigma‐Aldrich unless otherwise specified. All vesicles were composed of POPC unless otherwise stated. All fluorescent lipids were headgroup labelled. All aqueous phases contained DI water. Lipid films were prepared by depositing lipid dissolved in chloroform in a glass vial, removing the chloroform under a stream of nitrogen, and placing the vial in a lyophiliser for 30 min. The lipid was then dissolved in oil to give a 2 mg mL^−1^ solution by sonication at 50 °C for 30 min.

To form two‐layered vesicles, an water/oil/water column was first prepared in an 1.5 mL Eppendorf, which consisted from bottom to top of sucrose (0.5 M; 300 μL), silicone oil (0.5 M; 500 μL), and glucose (0.5 M; 200 μL). This column was left to stabilise for 30 min to allow lipid monolayers to form at the water/oil interfaces. A water‐in‐oil emulsion was then prepared by vigorously aspirating up and down, with a pipette, a mixture of maltoriose (10 μL) in water and mineral oil containing dissolved lipid (90 μL). This emulsion was layered on the top of the column, which was then placed in a centrifuge (Eppendorf centrifuge 5415D; 9000 *g*, 30 min), yielding a vesicle pellet. The supernatant was removed, and the pellet was resuspended in sucrose (200 μL), followed by a second centrifugation and resuspension step, yielding a solution containing two‐layered vesicles.

To generate three‐layered vesicles, two‐layered vesicles were prepared as above, but with the pellet resuspended in 200 μL glucose in the final step. This was then deposited on a second pre‐prepared column consisting of sucrose (0.5 M; 300 μL), silicone oil AR 200 (500 μL), and glucose (0.5 M; 200 μL), which was left to stabilise for 30 min. Subsequent steps were identical to those used in two‐layered vesicle production.

**Imaging and microscopy**: Vesicles were imaged by diluting the sample in 0.5 M sucrose (1 : 9), adding 50 ul of this solution to a PDMS spacer (1 mm thick, 10 mm diameter) on a microscopy slide, and sealing the chamber with a cover slip. Vesicles were visualised with a Nikon Eclipse TE2000‐E inverted microscope with phase contrast objectives. Images and video were captured with a Ximea MQ013MG‐E2 camera. Fluorescence imaging experiments used an illuminating mercury arc lamp, with FITC, TRITC, and Cy‐5 filters used to image NBD, rhodamine, and Cy‐5 fluorescent moieties respectively.

**Multilamellar vesicles validation (intensities and quencher experiments)**: The fluorescence intensity of single‐, double‐, and triple‐layered vesicles composed of POPC with 1 % Rh‐PE in all bilayers was determined by analysing the fluorescence of vesicles 5 μm radius or larger. The microscope was focussed to image the vesicle equator, and florescent images acquired (TRITC filter; 50 ms exposure). The average fluorescence per unit area of the entire vesicle was determined using the mean grey value function in ImageJ (NIH) software. For the external bilayer quenching experiments, two‐layered POPC vesicles were manufactured with NBD‐PE in the outer or inner bilayers. Vesicles were diluted 1 : 9 in sucrose (0.5 M), followed by a 9 : 1 dilution with sodium dithionate (0.5 M) in sucrose (0.5 M), to yield a final quencher concentration of 0.05 M. The solution was mixed by pipetting up and down five times, and NBD fluorescence was imaged with a fluorescence microscope (FITC filter; 300 ms exposure) within 5 min. Analysis was conducted by extracting the membrane contour fluorescence intensity value on ImageJ, and normalising the data with respect to the fluorescence intensity at the first image acquisition point.

**Domain alignment**: Two‐layered vesicles composed of DPhPC/EggSM/cholesterol 1 : 1 : 3 were manufactured as described above, with 1 wt % NBD‐PE in the inner layer and 1 wt % Rh‐PE in the outer layer. This composition (with high levels of cholesterol), was used to compensate for the inefficient incorporation of cholesterol in the emulsion phase transfer technique.^[34]^ NBD‐PE and Rh‐PE fluorescent lipids were imaged using FITC and TRITC filters with 500 and 50 ms exposure times respectively. To track domain alignment during mixing and demixing, vesicles were heated past their transition temperature using a heating stage attached to the microscope, and then left to cool to room temperature.

**Flickering spectroscopy**: Flickering spectroscopy was conducted as described previously.^[26]^ Briefly, 60 s videos were recorded at a frame rate of 120 fps and an exposure time of 0.4 ms. Data analysis was carried out using a custom‐built LabVIEW (National Instruments) program that detects and extracts membrane contours from each frame with subpixel resolution. Full details of membrane flickering analysis are given elsewhere.^[28]^ Briefly, the deviation of each contour from the mean membrane position was decomposed into fluctuation modes by Fourier transforming to give a fluctuation power spectrum of mean square mode amplitudes at the cell equator [*h* ^2^(*q_x_*, *y*=0)] as a function of mode wavenumber (*q_x_*). From these data, the bending modulus (*κ*) and tension (*σ*) can be fitted using the following equation:
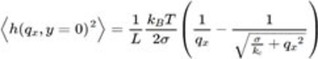



where *k*_B_ is the Boltzmann constant, *T* is temperature, and *L* is mean circumference of the vesicle contour. This model assumes that the cell surface behaves as a flat sheet and that we image the equator of the cell. When fitting the fluctuation data, mode numbers 4 and below were excluded due to significant influence of the vesicle shape (breakdown of the flat sheet assumption) and mode numbers above 20 were removed because these fluctuations lie outside the spatial and temporal resolution of the experiment. The bending rigidity was resolved using a double‐parameter nonlinear fit to the model above. Only vesicles which were free of visible debris, aggregates, and deformations were analysed. Vesicles which were larger than 5 μm in diameter and which exhibited a non‐homogenous signal intensity when imaged under fluorescence mode were not analysed.

## Conflict of interest

The authors declare no conflict of interest.

## Supporting information

As a service to our authors and readers, this journal provides supporting information supplied by the authors. Such materials are peer reviewed and may be re‐organized for online delivery, but are not copy‐edited or typeset. Technical support issues arising from supporting information (other than missing files) should be addressed to the authors.

Supplementary
