# Supplementary material for: Manufacture of Multilayered Artificial Cell Membranes through Sequential Bilayer Deposition on Emulsion Templates
Source: Chembiochem. 2021 Mar 31;22(13):2275–81. doi: 10.1002/cbic.202100072 (PMC8360201; doi:10.1002/cbic.202100072)

ChemBioChem

Supporting Information

Manufacture of Multilayered Artificial Cell Membranes through Sequential Bilayer Deposition on Emulsion Templates

Tsoi Ip[+], Qien Li[+], Nick Brooks, and Yuval Elani*

Supplementary Figure 1: Histogram of multi-layered vesicle diameters

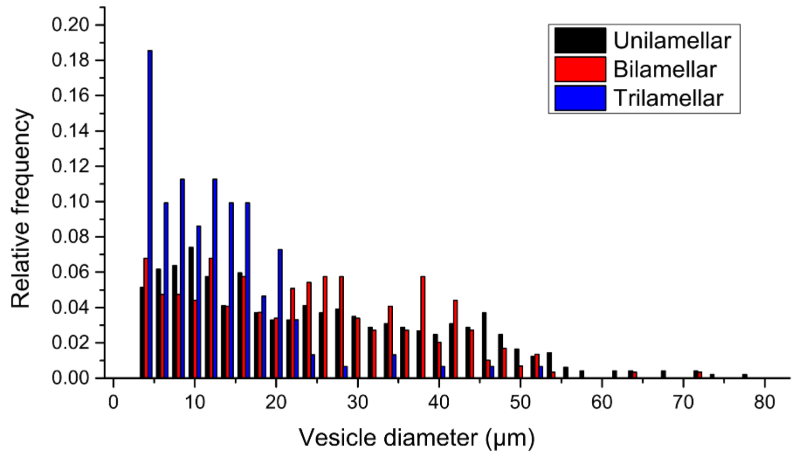

Supplementary Figure 2: Fluctuation analysis fitting for the determination of bending rigidity. Representative spectra of individual (a) one- (b) two- and (c) three-layered vesicles. The log of the mean square amplitude of contour fluctuation modes versus the log of the wave vector is plotted. The graph is fitted to the equation described in the methods to extract the bending modulus (κ) (blue line). Full details are provided by Yoon et al (Biophysical journal 97, 1606-1615, 2009). The vesicle shape is first extracted for each frame in a video by radially integrating the image intensity. The contour is then Fourier-transformed to break down the contour shape into individual fluctuation modes and their associated amplitudes.

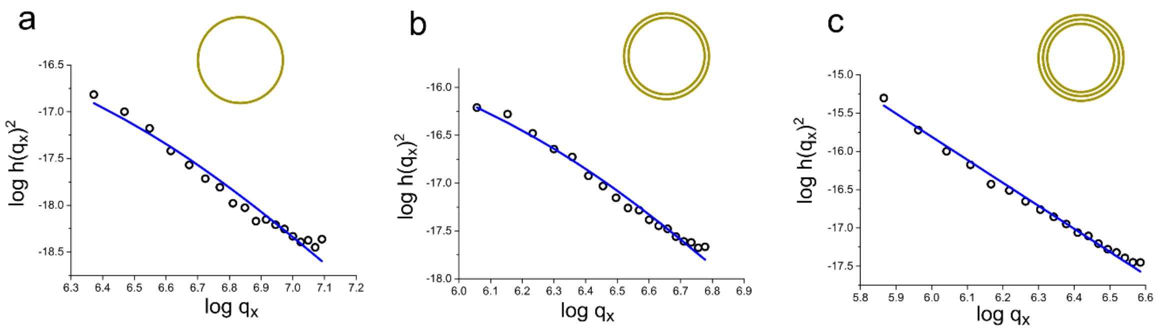

Supplement: Supplementary file 1 — Supplementary [file CBIC-22-2275-s001.pdf]
